# Quantification of Seventeen Phenolic Acids in Non-Soy Tempeh Alternatives Based on Legumes, Pseudocereals, and Cereals

**DOI:** 10.3390/foods14132273

**Published:** 2025-06-26

**Authors:** Miloslav Šulc, Jana Rysová

**Affiliations:** Czech Agrifood Research Center, Food Science Group, Radiova 1285/7, Praha 10—Hostivar, 10200 Prague, Czech Republic; jana.rysova@carc.cz

**Keywords:** phenolic acid, phenolic compound, tempeh, solid state fermentation, sorghum, proso millet, bean, buckwheat, pea, quinoa

## Abstract

The rising demand for sustainable and health-promoting foods has encouraged the development of tempeh from non-soy plant materials. This study investigated tempeh alternatives made from sorghum, proso millet, white bean, buckwheat, yellow pea, and quinoa, focusing on their phenolic acid (PA) content. Seventeen PAs and two flavan-3-ols were quantified using LC-MS/MS in free, conjugated, and insoluble forms, and total phenolic content (TPC) was determined using the Folin–Ciocalteu assay. Four PAs—shikimic acid, 3-hydroxycinnamic acid, 3,5-dihydroxybenzoic acid, and 2-hydroxycinnamic acid—were not detected. Solid-state fermentation increased the total PA (TPA) content by an average of 11.3%, reaching 160.6 µg/g, with the most significant rise in conjugated and insoluble fractions. The highest TPA values were observed in sorghum-based tempeh, particularly quinoa:sorghum (2:1; 293 µg/g), sorghum:yellow pea (2:1; 277.6 µg/g), and buckwheat:sorghum (1:1; 271 µg/g). The most abundant PAs were ferulic (18 µg/g), vanillic (14.6 µg/g), 3,4-dihydroxybenzoic (8 µg/g), and caffeic acids (6.7 µg/g). TPC values reached up to 9.51 mg GAE/g in tempeh samples. These findings support the use of non-soy substrates to develop nutritious, allergen-free, gluten-free tempeh products with enhanced phenolic profiles and functional food potential.

## 1. Introduction

Tempeh is a traditional Indonesian fermented food in which *Rhizopus* fungi and soybeans play the essential role [1]. Due to its good nutritional value, tempeh is an excellent source of high-quality, low-cost protein, thus serving as an alternative to meat. Tempeh has also the advantage of containing vitamin B_12_, which is a by-product of solid-state fermentation (SSF) [2]. Tempeh production is relatively simple and consists of soaking (8–24 h), dehulling, boiling (1 h), and fermenting (30–72 h), which produce chemical changes (increase in soluble proteins and soluble carbohydrates, decrease in antinutritional factors, like protease inhibitors, phytic acid, tannin content, and flatulence producing factors) [1,3]. In addition, the presence of phenolic compounds in tempeh may help improve its functional properties [4]. Tempeh is traditionally made from soybeans but other plant materials might also be used: grass pea [5], jojoba seed [1], rice bran [4], buckwheat [6], spelt [7], *Moringa* seeds [8], corn [9], millet [10], Jack bean [11], kidney bean [12], quinoa [13], and many more [14].

Solid-state fermentation (SSF) with microorganism is an economically viable method used to biotransform food materials for nutritional enrichment. Tempeh is traditionally produced with strains such as *Rhizopus oligosporus*, *R. arrhizus*, or *R. stolonifer* [15]. SSF represents a technological alternative for processing a great variety of legumes and/or cereals to improve their nutritional and nutraceutical properties and to produce edible products with palatable sensory characteristics. An important function of the fungus during fermentation is the synthesis of enzymes, which hydrolyze some of the substrate constituents and contribute to the development of a desirable texture, flavor, and aroma of the product. Consequently, the nutritional quality of the fermented food may be improved [16].

Phenolic acids are secondary plant metabolites and bioactive chemicals grouped under phenolic compounds occurring in various plant sources (fruits, seeds, leaves, and roots). Phenolic acids have immense dietary health benefits and functionalities, like antioxidant, anti-inflammatory, immunoregulatory, anti-allergic, anti-atherogenic, anti-microbial, anti-thrombotic, cardioprotective, anti-cancer, and antidiabetic properties [17]. Phenolic acids are comprised of one-third of the constituents among phenolic compounds and occur in three forms: (a) free, (b) conjugated (linked to sugars, organic acids, or low-molecular-weight compounds), (c) insoluble (covalently bonded to lignin, cellulose, or proteins) [18]. They are classified as hydroxybenzoic acids (C6–C1) and hydroxycinnamic acids (C6–C3) because of their two distinctive carbon frameworks, as well as depending on the positioning and number of the hydroxyl groups on the aromatic ring. Hydroxycinnamic acids are more prevalent in nature than hydroxybenzoic acids [19]. In plant seeds, polyphenols are primarily located in the outer layers, where they play a protective role against environmental stress, pathogens, and predators. Since polyphenols are mainly concentrated in the outer layers, refining processes (such as polishing, milling, or dehulling) often reduce their content in foods like white rice and refined flour [20,21]. Fermentation, as in tempeh production, may help release bound polyphenols, enhancing their bioavailability [22,23].

Recent studies demonstrate that tempeh fermentation using non-soy substrates can significantly modulate PA profiles and antioxidant activity. SSF with *Rhizopus* spp. increases extractable PAs in cereals such as spelt, corn, and buckwheat, with ferulic acid emerging as the most responsive compound due to microbial enzymatic release from bound forms [7,9]. In legumes, including grass pea, pea, and kidney bean, fermentation often enhances free PA levels and antioxidant potential, especially when co-fermented with *Lactobacillus plantarum* [5]. Quinoa-based tempeh showed increased soluble phenolics and a higher radical scavenging capacity compared to raw grains, despite differences across seed color variants [24]. Similarly, tempeh from millet and bean mixtures exhibited improved nutritional and sensory attributes, including increased protein and phenolics content [10,11]. However, fermentation outcomes depend on substrate type, fermentation time, and microbial strains. While free phenolic content may initially decrease during soaking and cooking, SSF generally enhances the total PA content, particularly in conjugated and bound forms, suggesting its effectiveness in upgrading the functional profile of alternative plant-based tempeh.

The primary purpose of this project was to develop new plant-based alternatives for tempeh production, particularly in regions where soybeans are not cultivated or where soybean consumption is low due to agricultural limitations or cultural dietary habits. Additionally, this project aims to offer suitable options for individuals with soy allergies or gluten-related health conditions, which affect nearly 5% of the global population [25], thereby expanding their available food choices. Additionally, factors such as concerns over glyphosate use in soy cultivation, genetically-modified (GMO) soy avoidance, and consumer interest in diverse plant-based meat alternatives further drive the need for the exploration of tempeh alternatives. Our study aimed to develop and evaluate tempeh alternatives using a diverse selection of legumes and (pseudo)cereals. While previous research has investigated PA content in all sorts of tempeh (mainly by Folin assay or HPLC), our study focused on a systematic analysis of a broad range of non-soy substrates and mixes thereof, many of which have not been previously characterized for their free, conjugated, and insoluble PA fractions, to quantify 17 phenolic acids and two flavan-3-ols across all samples; and to compare the results with the published data on traditional soy and non-soy tempeh to assess the functional potential of these alternatives. By doing so, we provide a detailed characterization of underutilized crops and contribute to the development of nutritionally valuable, sustainable, and inclusive tempeh products.

## 2. Materials and Methods

### 2.1. Chemicals

Glacial acetic acid (ACS grade, Merck, Praha, Czechia), hydrochloric acid (35%, ACS grade, Lachner, Neratovice, Czechia), sodium hydroxide (ACS grade, Lachner, Neratovice, Czechia), sodium chloride (ACS grade, Lachner, Neratovice, Czechia), compressed nitrogen (cylinder, 20 L, 200 bar, ≥99.99%, Linde, Praha, Czechia), water for chromatography (see Section 2.2), ascorbic acid (ACS grade, Merck, Praha, Czechia), butylated hydroxyanisole (BHA, ACS grade, Jan Dekker, Amsterdam, The Netherlands), ethylenediaminetetraacetic acid disodium salt dihydrate (EDTA-K_2_, ACS grade, Merck, Praha, Czechia), petroleum ether (40–65 °C, >90%, ACS grade, Lachner, Neratovice, Czechia), acetonitrile (for chromatography/HPLC grade, Fisher, Pardubice, Czechia), internal standard: 3,5-dichloro-4-hydroxybenzoic acid (97%, Merck, Praha, Czechia), Folin and Ciocalteu’s phenol reagent (Sigma-Aldrich/Merck, Praha, Czechia), sodium carbonate anhydrous (ACS grade, Lachner, Neratovice, Czechia), and ethanol (96%, denatured with 1% petroleum ether; Lachner, Neratovice, Czechia).

All 19 analytical standards of phenolic compounds ((+)-catechin, (−)-epicatechin, 2,5-dihydroxybenzoic acid, 2-hydroxycinnamic acid, 3,4-dihydroxybenzoic acid, 3,5-dihydroxybenzoic acid, 3-hydroxybenzoic acid, 3-hydroxycinnamic acid, 4-hydroxybenzoic acid, 4-hydroxycinnamic acid, caffeic acid, chlorogenic acid, ferulic acid, gallic acid, salicylic acid, shikimic acid, sinapic acid, syringic acid, and vanillic acid) were purchased from Merck (Praha, Czechia).

### 2.2. Apparatuses

An Agilent 6460 triple quadrupole MS coupled to Agilent 1260 Infinity II HPLC comprising a binary pump, column oven, and vial sampler (Waldbronn, Germany) was used for chromatographic analysis and data acquisition. The Milli-Q water purification system (>18.2 MΩ) by Merck/Millipore (Praha, Czechia), chromatographic columns: Agilent Poroshell 120 EC-C18: 2.7 µm 3 × 150 mm, with a guard column Agilent Poroshell 120 EC-C18: 5 × 3 mm 2.7 µm), a nitrogen evaporator Mini Dry Bath by Hangzhou Miu Instruments (Hangzhou, China), a centrifuge 352 R with rotors for 15 and 50 mL centrifuge tubes by MPW Med. Instruments (Warsaw, Poland), a rotary evaporator system by Büchi (controller V850, rotavapor R-210, bath B-411, vacuum pump V-700, and chiller F-105, Flawil, Switzerland), linear shaker Heidolph Promax 1020 (Schwabach, Germany), ultrasonic probe Bandelin electronic DH2200, ultrasonic bath Sonorex 35 kHz RK 225 H by Bandelin (Berlin, Germany), convection drying oven ED 115 by Binder (Tuttlingen, Germany), UV/VIS spectrophotometer Helios α by Thermo Scientific (Praha, Czechia), 1 cm polypropylene cuvettes by Brand (Wertheim, Germany), water bath model 1042 by GFL (Berlin, Germany), and an incubator (for microbiology) BT120 by Laboratorni pristroje Praha (Praha, Czechia).

### 2.3. Plant Material

The plant material (3 kg each) used for tempeh production was obtained from retail sources as dried seeds in Prague, Czech Republic, in 2024. Sorghum (*Sorghum bicolor* L.) peeled; proso millet (*Panicum miliaceum* L.) peeled; common bean (*Phaseolus vulgaris* L.) white; buckwheat (*Fagopyrum esculentum* Moench) peeled; pea (*Pisum sativum* L.) yellow, split, and peeled (dehulled); and quinoa (*Chenopodium quinoa* Willd.) white were used.

### 2.4. Tempeh Production (Solid-State Fermentation)

**Figure 1:**
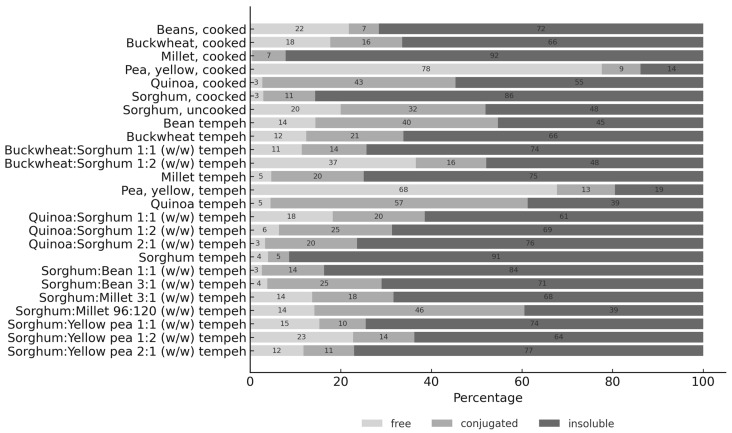
Normalized PA composition (as % of total PA) in raw materials and tempeh samples (values rounded to whole numbers).

White beans were soaked in water overnight and manually dehulled prior to thermal processing. Then, soaked, dehulled white beans and any other dry seeds were cooked to a semi-soft consistency, drained, and cooled to room temperature. Only beans and peas were coarsely ground to a particle size of approximately 3–4 mm. The cooked seeds were used as is or blended in the desired proportions by weight (see result tables) and inoculated with a starter culture (*Rhizopus oligosporus* cultivated on rice flour, Ragi Tempe, Raprima, Indonesia) at a concentration of 1 g per kg of substrate. The inoculated substrate was transferred into perforated polyethylene sleeves (to ensure adequate oxygen availability during fermentation), which were sealed at both ends. The fermentation was carried out for 38 h (30 °C, 90% relative humidity) in an incubator on a wire rack. After fermentation, the tempeh was removed from the polyethylene sleeves (Figure 1), finely crumbled by hand, and dried at 75 °C to a constant weight (about 20 h), then finely ground and stored in polypropylene containers in a refrigerator (4 °C) for subsequent analyses. Each tempeh sample was prepared in triplicates fermented on different days within one month. Selected tempeh photographs can be found in the Supplementary Materials.

### 2.5. Defatting

A total of 15 g of the dried tempeh sample was weighed into a cellulose extraction thimble and extracted with 200 mL of petroleum ether for 4 h in Soxhlet apparatus at 70 °C. The thimbles were removed and left to completely evaporate the solvent overnight in a fume hood.

### 2.6. Extractions

The extraction of phenolic acids in three separated forms (free, conjugated, and insoluble) was performed in triplicate according to [26,27,28] with modifications.

A total of 1 g of defatted tempeh sample or raw material was weighed into a 15 mL polypropylene (PP) centrifuge tube and 4 mL of extraction solvent (consisting of 10 mL of glacial acetic acid, 2 g of BHA, 3.33 mg of IS, and methanol as a solvent per 100 mL). The tubes were shaken on a linear shaker for 45 min (250 rpm), then sonicated in an ultrasonic bath for 10 min, and finally centrifuged for 10 min at 10,000 rpm. The clear supernatant was carefully decanted into another 15 mL PP centrifuge tube. The extraction was repeated three times in total and all the supernatants pooled. The pooled methanol extracts were used: one part used for the determination of free PA (Section 2.6.1), another for conjugated PA (Section 2.6.2).

#### 2.6.1. Free Phenolic Acids

A total of 3 mL of pooled methanolic extract (Section 2.6) was pipetted into a 5 mL PP centrifuge tube and evaporated with nitrogen for 35 min at 70 °C to dryness. Dried samples were stored in a refrigerator (4 °C) overnight. On the next day, 1.5 mL of 2% aqueous formic acid was added to the dry sample and the dry residue was dissolved and disintegrated by an ultrasonic probe (about 20 pulses, 30% cycle, and 70% power), and the sample was vortexed twice for 5 s. Then, the sample was acidified with 5.3 µL 35% HCl (lowering pH < 2) and vortexed again twice for 5 s. Next, 1.5 mL of ethyl acetate was added and the mixture vortexed twice for 5 s and shaken on a linear shaker for 30 min to extract it. Then, the sample was centrifuged for 10 min at 7000 rpm to separate the two layers. The upper (ethylacetate) layer was then transferred by a plastic Pasteur pipette into another 5 mL PP centrifuge tube, ethylacetate extraction was repeated two more times, and the three ethylacetate extracts were pooled and evaporated under nitrogen for 35 min at 70 °C. Dried samples were stored in a refrigerator (4 °C) overnight. On the next day, the dried sample was redissolved in 1 mL of 10% acetic acid in methanol, vortexed twice for 5 s, and filtered through a syringe filter with a nylon membrane (0.22 µm) into a clear-glass HPLC vial with a PTFE/silicone cap. Samples were prepared in triplicates.

#### 2.6.2. Conjugated Phenolic Acids

A total of 3 mL of pooled methanolic extract (Section 2.6) was pipetted into a 5 mL PP centrifuge tube and evaporated with nitrogen for 35 min at 70 °C to dryness. Dried samples were stored in a refrigerator (4 °C) overnight. On the next day, 1.25 mL of water was added to the dry residue, dissolved and disintegrated by an ultrasonic probe (about 20 pulses, 30% cycle, and 70% power), and the sample was vortexed twice for 5 s. Then, 1.25 mL of 4 M aqueous sodium hydroxide was added and vortexed twice for 5 s. The sample was hydrolyzed for 4 h at room temperature in the dark on a linear shaker. After hydrolysis, the sample was acidified with 440 µL 35% HCl (lowering pH < 2) and vortexed again twice for 5 s. Next, 1.5 mL of ethyl acetate was added and the mixture was vortexed twice for 5 s and shaken on a linear shaker for 30 min to extract it. Then, the sample was centrifuged for 10 min at 7000 rpm to separate the two layers. The upper (ethylacetate) layer was then transferred by a plastic Pasteur pipette into another 5 mL PP centrifuge tube, ethylacetate extraction was repeated two more times, and the three ethylacetate extracts were pooled and evaporated under nitrogen for 35 min at 70 °C. Dried samples were stored in a refrigerator (4 °C) overnight. On the next day, the dried sample was redissolved in 1 mL of 10% acetic acid in methanol, vortexed twice for 5 s, and filtered through a syringe filter with a nylon membrane (0.22 µm) into a clear-glass HPLC vial with a PTFE/silicone cap. Samples were prepared in triplicates.

#### 2.6.3. Insoluble (Bound) Phenolic Acids

A total of 1 g of the defatted tempeh sample or raw material was weighted into 50 mL polypropylene (PP) centrifuge tube and 4 mL of extraction solvent (consisting of 10 mL of glacial acetic acid, 2 g of BHA, and methanol as a solvent per 100 mL). The tubes were shaken on a linear shaker for 45 min (250 rpm), then sonicated in an ultrasonic bath for 10 min, and finally centrifuged for 10 min at 10,000 rpm. The supernatant was discarded. The extraction was repeated three times in total, keeping only the pellet at the end. The remaining residual solvent from the pellet was evaporated at laboratory temperature overnight. On the next day, the pellet was disintegrated with a glass rod and 100 µL of IS (1 mg/mL in methanol) was added. Then, 12.5 mL of water containing 1% (*w*/*v*) ascorbic acid and 0.5% (*w*/*v*) EDTA-K_2_ was shaken vigorously by hand to disintegrate the pellet into the solution. Next, 12.5 mL of 4 M aqueous sodium hydroxide solution added and immediately shaken vigorously by hand. The sample was hydrolyzed for 4 h at room temperature by shaking on a linear shaker. To stop the hydrolysis, the sample was acidified with 4.3 mL of 35% HCl (lowering pH < 2) and vortexed again twice for 5 s. Then, 5 g (±10%) of sodium chloride was added and the sample shaken for 5 min on a linear shaker to dissolve it. Next, 10 mL of ethyl acetate was added and the mixture vortexed twice for 5 s and shaken on linear shaker for 30 min to extract it. Then, the sample was centrifuged for 15 min at 10,000 rpm to separate the two layers. The upper (ethylacetate) layer was then transferred by a plastic Pasteur pipette into a 50 mL glass round-bottom evaporation flask, and ethylacetate extraction was repeated two more times (pooling the three ethylacetate extracts). The entire volume of the pooled extract was evaporated under vacuum in a rotary evaporator for 8 min (bath temperature: 50 °C, cooling medium: −7 °C, and vacuum at 50 mbar). Dried samples were stored in a refrigerator (4 °C) overnight. On the next day, the dried sample was redissolved in 1 mL of 10% acetic acid in methanol, vortexed twice for 5 s, and filtered through a syringe filter with a nylon membrane (0.22 µm) into a clear-glass HPLC vial with a PTFE/silicone cap. Samples were prepared in triplicates.

### 2.7. LC-MS/MS

The binary mobile phase consisted of 0.25% (*v*/*v*) formic acid in acetonitrile (A) and 0.25% (*v*/*v*) formic acid in water. The injection volume was 1 µL, column oven temperature 30 °C, autosampler temperature 10 °C; for the LC-MS/MS type, column, and guard column, see Section 2.2. The mobile phase flow was 0.5 mL/min; the needle was rinsed in port before injection for 5 s with acetonitrile:water, 50:50 (*v*/*v*); the total run time was 30 min; and the gradient elution values were 0 min 5% A, 15 min 60% A, 17 min 90% A, 22 min 90% A, 23 min 5% A, and 30 min 5% A. MRM transitions in the negative ionization mode were collected from 0 to 12 min; the MS source parameters were gas temp. 350 °C, gas flow 8 L/min, nebulizer 30 psi, sheath gas temp. 350 °C, sheath gas flow 12 L/min, capillary 3000 V, and nozzle 600 V. MS1 were and MS2 in unit resolution, the dwell time was 10 ms, and the cell accelerator operated a 4 V. Precursor and fragment ions with details are shown in Appendix B (Table A2). A calibration stock solution of all analytes (0.1 mg/mL per analyte in methanol containing 2% BHA) was prepared, portioned into aliquots, and stored at −40 °C for 2 months. Five-point calibration curve values of 0.5, 10, 30, 50, and 70 µg/mL per analyte were made and 1/x weighing applied. Each calibration point included IS (3,5-dichloro-4-hydroxybenzoic acid) with a concentration of 100 µg/mL. The results are calculated as µg/gram of sample (DW). Method validation data as well as LC-MS/MS chromatograms in the TIC mode and analyte retention times can be found in the Supplementary Materials.

### 2.8. Total Phenolic Content (TPC)

The method is based on [29] with the following modifications: 1–3 g of dried tempeh or raw material was extracted in 100 mL of 80% ethanol at 85 °C for 60 min in a shaking water bath (with an attached cooler). After cooling, 3 mL of the solution was filtered through a 0.45 µm nylon membrane syringe filter. A total of 1 mL of the filtrate was transferred into a 50 mL volumetric flask containing 25 mL of distilled water. Subsequently, 2.5 mL of Folin and Ciocalteu’s phenol reagent and 7.5 mL of 20% (*w*/*v*) aqueous sodium carbonate solution were added, mixed, and filled up to the mark with distilled water. After standing for 2 h, the absorbance was measured at 765 nm in 10 mm PP cuvettes against a blank. A 5-point calibration curve (20–250 µg/mL) using gallic acid as a standard was made, and the sample results are reported as gallic acid equivalents (GAEs) in mg/g of sample (DW).

### 2.9. Data Processing

For LC-MS/MS data processing, the Agilent MassHunter software package (version B08.02) was used. Statistical analysis and graphical outputs were prepared using Statistica 12 (StatSoft) and Microsoft Excel (version 2505).

## 3. Results

This study analyzed the concentrations of free, conjugated, and insoluble phenolic acids (PAs) in both raw materials and final products across various tempeh alternatives. Regardless of the raw material source, the average total PA concentration in the raw substrates was 144.3 µg/g, compared to 160.6 µg/g in the corresponding tempeh products. This reflects an average increase of 11.3% in PA availability following solid-state fermentation (SSF), as shown in Table 1. An increase was observed in both conjugated and insoluble PAs, while free PA concentrations declined during fermentation. Across all samples, insoluble PAs were the predominant fraction, followed by conjugated and then free PAs (Figure 1).

The seven samples (Table 2) with the highest total PA content were all tempeh products, ranging from 293 µg/g in the quinoa:sorghum (2:1) tempeh to 186.2 µg/g in the sorghum:bean (1:1) tempeh. Conversely, the lowest total PA levels were found in cooked beans (50.9 µg/g), followed by bean tempeh (52.4 µg/g) and sorghum:millet (3:1) tempeh (83.5 µg/g).

The raw materials with the highest total phenolic acid (TPA) content were millet (179.2 µg/g TPA), quinoa (176.9 µg/g TPA), yellow pea (164.5 µg/g TPA), and sorghum (130.4 µg/g TPA). As shown in Figure 1, the highest concentrations of free phenolic acids were found in cooked yellow pea (127.7 µg/g, representing 78% of total PA), buckwheat:sorghum (1:2) tempeh (75.5 µg/g, 37% of TPA), and yellow pea tempeh (59.5 µg/g, 68% of TPA). Conjugated phenolic acids were most abundant in the quinoa tempeh (127.7 µg/g, 57% of TPA), cooked quinoa (75.5 µg/g, 43% of TPA), and quinoa:sorghum (2:1) tempeh (59.5 µg/g, 20% of TPA). The materials with the highest levels of insoluble phenolic acids were quinoa:sorghum (2:1) tempeh (223.9 µg/g, 76% of TPA), sorghum:yellow pea (2:1) tempeh (214 µg/g, 77% of TPA), and buckwheat:sorghum (1:1) tempeh (201.5 µg/g, 74% of TPA).

The individual contents of all nineteen analytes in their free, conjugated, and insoluble forms are listed in Appendix A (Table A1). Four phenolic acids were never detected in any of the samples: shikimic acid, 3-hydroxycinnamic acid, 3,5-dihydroxybenzoic acid, and 2-hydroxycinnamic acid. These were followed by the least abundant PAs detected in the dataset, which included 3-hydroxybenzoic acid, chlorogenic acid, and syringic acid. Based on the average content per sample, the most abundant PAs in the raw materials were ferulic acid (48.8 µg/g), 3,4-dihydroxybenzoic acid (21.5 µg/g), caffeic acid (15 µg/g), and vanillic acid (14.6 µg/g). For obvious reasons, these same PAs were also, on average, the most abundant in tempeh samples, although their concentrations varied due to the blending of different raw materials. When considering the total concentration of each individual PA across all samples, the most abundant compounds were ferulic acid (∑ = 1559.4 µg/g), 3,4-dihydroxybenzoic acid (∑ = 512.3 µg/g), caffeic acid (∑ = 470.3 µg/g), and 4-hydroxycinnamic acid (∑ = 352.9 µg/g). Examining individual cases, the highest ferulic acid content was found in quinoa:sorghum (2:1) tempeh (185.4 µg/g), the highest vanillic acid in quinoa tempeh (88.1 µg/g), the highest 3,4-dihydroxybenzoic acid in uncooked sorghum (63 µg/g), and the highest caffeic acid also in uncooked sorghum (47.6 µg/g). Comparing two raw materials for which both uncooked and cooked states were evaluated, cooking resulted in a slight decrease in TPA content from 172.4 to 130.4 µg/g in sorghum.

Total phenolic content (TPC), measured using the Folin–Ciocalteu method, is presented in Table 2. The mean TPC for raw materials was 1.6 mg GAE/g, while the mean TPC for tempeh samples was 9.2 mg GAE/g. The LC-MS/MS results for individual PAs also show lower average concentrations in raw materials. However, the Pearson’s correlation coefficient between TPA and TPC was very weak (r = 0.11, *p* = 0.604), indicating a poor correlation between the two variables.

The only two phenolic compounds analyzed that were not classified as PAs were two stereoisomers of catechin (a flavan-3-ol), which are known structural units of tannins. These were chosen to assess the possible astringency of the product by sensory testing. Among these, (+)-catechin was more commonly detected, with its highest concentrations found in cooked buckwheat (16.0 µg/g) and uncooked sorghum (13.0 µg/g). The highest concentration of (−)-catechin was also observed in cooked buckwheat (7.2 µg/g).

## 4. Discussion

From a health perspective, traditional tempeh has been shown to exhibit anti-diabetic, cholesterol-lowering, antitumor, antihypertensive, and cognitive-enhancing effects, as well as properties that support gut health and alleviate depressive symptoms [30]. Some of these health benefits are attributed to bioactive compounds naturally present in soybeans, such as isoflavones. Similarly, in tempeh alternatives, health-promoting effects are linked either to the substrate used (e.g., quercetin and rutin in buckwheat) or to the fungal inoculum employed during SSF. *Rhizopus* spp. has been reported to enhance the bioavailability of macro- and micronutrients, including amino acids, vitamins, phenolic compounds, S-adenosylmethionine, and β-nicotinamide mononucleotide [31]. It also contributes to gut health by increasing levels of short-chain fatty acids (acetate and propionate), stimulating mucin production, and positively modulating gut microbiota composition [32]. Additionally, *Rhizopus* spp. possesses antioxidant and anticancer activities [33]. The research has demonstrated that high-quality tempeh can be produced from legumes other than soybean [34]. Moreover, *Rhizopus* spp. is capable of reducing antinutritional compounds present in the substrates, such as oxalates and phytate [35].

Phenolic acids are secondary plant metabolites that contribute to the color, flavor, and astringency of foods, playing a significant role in defining their organoleptic characteristics. The primary value of PAs lies in their health-related properties [19]. The content of PA in foods and plants varies significantly depending on the specific type of food or plant, the part of the plant, growing conditions, processing methods, and even the variety or cultivar and year [36]. Furthermore, variability in reported PA content is influenced by the plant material used, the processing it undergoes, the analytical methods applied, and whether the PAs are reported as free, bound, or total. Typically, PA content ranges from 1 to 300 mg per 100 g of fresh weight (FW) [37,38,39]. Studies have shown that legume hulls contain higher concentrations of PAs compared to dehulled seeds [20,21,40]. Estimates of daily PA intake range from 158 to 1266 mg, with coffee alone accounting for approximately 55–80% of total intake, followed by fruits, vegetables, and nuts. Hydroxycinnamic acids represent the predominant class of dietary PAs, comprising 85–95% of overall intake. This predominance is due to their biosynthesis via the highly active phenylpropanoid pathway in plants, which also leads to lignin production. In contrast, hydroxybenzoic acids are primarily formed through the degradation of lignin and tannins, and therefore accumulate to a lesser extent [41].

In our study, the average TPA content in tempeh samples was relatively low (160.6 µg/g), which can be attributed to the fact that all plant materials used for tempeh production were either dehulled or polished (except for quinoa where whole seeds were used). These preprocessing treatments are known to reduce the phenolic content in raw materials [20], as the majority of phenolic compounds are concentrated in the outer seed layers that are removed during tempeh preparation. Additionally, soaking and cooking processes have been shown to further decrease the phenolic content by 15–80%, depending on the type of seed and the processing method used [2,3,42,43]. This reduction occurs because many phenolic compounds are water-soluble and are leached out during these treatments. For example, in our data, sorghum showed a decrease in TPA from 172.4 µg/g in raw seeds to 130.4 µg/g in cooked seeds. Similarly, the average concentration of free PAs—being the most water-soluble fraction—dropped from 28.0 µg/g in raw materials to 21.2 µg/g in tempeh samples. Together, these two factors—dehulling/polishing and cooking—account for the generally low PA levels observed in the starting materials used to produce tempeh alternatives, as most phenolics are removed during pretreatment. On the other hand, *Rhizopus* spp. has demonstrated the ability to enhance phenolic content due to its β-glucosidase enzymatic activity, which is able to hydrolyze glycosides and release phenolic aglycones during SSF [2]. This effect is evident in the increased TPA observed in tempeh compared to their respective cooked substrates: cooked beans versus bean tempeh (50.9 µg/g→52.4 µg/g, +2.9%), cooked millet versus millet tempeh (179.2 µg/g→202.3 µg/g, +12.8%), cooked buckwheat versus buckwheat tempeh (132.6 µg/g→147.4 µg/g, +11.2%), and cooked quinoa versus quinoa tempeh (176.9 µg/g→225.0 µg/g, +27.1%). On average, this corresponds to an overall increase of 11.3% in TPA when comparing tempeh samples to their respective raw materials.

The most abundant PAs identified in our non-soy raw materials were ferulic acid, vanillic acid, 3,4-dihydroxybenzoic acid, 4-hydroxycinnamic acid, and caffeic acid (Appendix A, Table A1). In comparison, a study by [44] using HPLC analysis reported the major PAs in soybeans as chlorogenic acid, 4-hydroxybenzoic acid, caffeic acid, ferulic acid, and gallic acid. Similarly, another study [42] analyzing PAs also by HPLC reported the major PAs found in thermally processed soybeans to be vanillic, gallic, sinapic, and cinnamic acid. A paper by [45] analyzed nine PAs in their free, conjugated, and bound forms across ten Brazilian soybean cultivars and reported average concentrations of 85.6 µg/g (DW) for free, 99.2 µg/g for conjugated, and 185.1 µg/g for insoluble PAs. These values are approximately three-times higher than those found in our non-soy raw materials, as shown in Table 1. This discrepancy may be explained by the following methodological differences: the cited study expressed values on a dry weight basis, used whole soybean seeds, and did not apply boiling or dehulling—treatments that are known to significantly reduce PA content. Regarding the fermentation time, it has been shown [15] that the content of both insoluble and soluble PAs (e.g., vanillic, syringic, and ferulic acids) in soybeans increases over the first 24 h of fermentation and subsequently decreases. Therefore, it is possible that our fermentation duration of 38 h may have contributed to a decline in PA content in the finished tempeh.

In buckwheat, the most predominant phenolic acids, as reported by [46,47], are ferulic, syringic, vanillic, p-coumaric, caffeic, and 3,4-dihydroxybenzoic acids. In our study, the major PAs identified were ferulic acid (∑ = 174.1 µg/g), caffeic acid (∑ = 110.4 µg/g), 4-hydroxybenzoic acid (∑ = 90.6 µg/g), and 3,4-dihydroxybenzoic (∑ = 87.0 µg/g). Certain acids showed notable increases during SSF, particularly caffeic acid, which rose from 6.6 µg/g to 22.7 µg/g, and ferulic acid, which increased from 4.7 µg/g to 17.6 µg/g. This increase was even more pronounced when buckwheat was combined with sorghum in the fermentation substrate—caffeic acid reached 42.8 µg/g in buckwheat:sorghum (1:2) tempeh, and ferulic acid rose to 130.9 µg/g in buckwheat:sorghum (1:1) tempeh.

In white bean tempeh, the TPA content increased only slightly by the activity of *Rhizopus* spp., from 50.9 µg/g (raw material) to 52.4 µg/g (tempeh). However, a significantly higher TPA content was observed in sorghum:bean (1:1) tempeh, which contained 186.2 µg/g TPA. According to [48], phenolic compounds are more concentrated in the seed hulls than in the cotyledons, particularly in red beans, with 4-hydroxycinnamic acid and ferulic acid being the dominant PAs—findings that are consistent with our results. The same study also identified catechin as one of the major phenolic compounds present in beans (that was not found in our samples using white beans). Another study [49] investigated unhulled, dark, common beans used for tempeh production and found that soaking and cooking led to a seven-fold decrease in total phenolic content, as measured by spectrophotometry. In contrast, a study by [16] reported a significant increase in phenolic content, from 2.83 to 6.09 mg catechin equivalents per gram. This discrepancy may be attributed to the analytical method used—the vanillin assay—which is susceptible to side reactions with non-phenolic compounds, potentially leading to an overestimation of phenolic content.

In a study of colored pea varieties analyzed by LC-MS, [50] 3,4-dihydroxybenzoic acid was identified as the most predominant PA, with concentrations ranging from 26 to 127 µg/g DW, and an average of 70.6 µg/g DW. This was followed by gallic acid (6.9 µg/g DW), ferulic acid (4.65 µg/g DW), and gentisic acid (3.9 µg/g DW). These findings are consistent with our results. Notably, the combination of sorghum and yellow pea in a 2:1 ratio yielded a particularly high TPA content (277.6 µg/g), suggesting it is a promising formulation for enhancing phenolic acid levels in tempeh.

Sorghum and proso millet are both cereal crops belonging to the *Poaceae* family. They share several adaptive traits, notably C4 photosynthesis, which enhances their water-use efficiency and makes them well-suited for cultivation in semi-arid and warm regions. Both crops are also gluten-free, making them increasingly important in health-conscious and allergen-sensitive diets. Sorghum has demonstrated potential as a promising base for tempeh production, particularly when combined with other ingredients that enhance PA content, such as quinoa (up to 293 µg/g TPA), yellow pea (up to 277.6 µg/g), and buckwheat (up to 271.0 µg/g). In a study by [51], 3,4-dihydroxybenzoic acid, caffeic acid, and ferulic acid were identified as the predominant PAs in wholegrain sorghum following hydromethanolic extraction. These findings align with our results, where sorghum—whether used alone or in combination—showed ferulic acid as the most abundant compound (∑ = 487.5 µg/g), followed by 3,4-dihydroxybenzoic acid (∑ = 233.5 µg/g), caffeic acid (∑ = 213.3 µg/g), and 4-hydroxycinnamic acid (∑ = 153.1 µg/g). In proso millet, the TPA content in tempeh increased slightly compared to the raw material, rising from 179.2 to 202.3 µg/g. However, millet blended with sorghum exhibited only average TPA levels among the tempeh varieties analyzed. The addition of sorghum to proso millet was effective in enhancing the concentration of several individual PAs in the resulting tempeh, including gallic acid, 3,4-dihydroxybenzoic acid, caffeic acid, 4-hydroxycinnamic acid, and sinapic acid (see Appendix A, Table A1).

Among the alternative seeds used for tempeh production, quinoa tempeh exhibited a notably high TPA content, reaching 225 µg/g. The highest TPA content was observed in quinoa:sorghum (2:1) tempeh, with 293.0 µg/g, indicating that quinoa can substantially enhance the PA concentration in tempeh compared to formulations based solely on beans. Ferulic acid was the most abundant PA in quinoa-based tempeh, with levels reaching up to 185.4 µg/g, consistent with the findings from previous studies on quinoa tempeh by [52].

To our knowledge, the four PAs not detected in our study—shikimic acid, 3-hydroxycinnamic acid, 3,5-dihydroxybenzoic acid, and 2-hydroxycinnamic acid—have also not been reported in the literature for buckwheat, millet, sorghum, peas, or beans.

To enable a comparison with values reported in the literature, total phenolic content (TPC) was measured in our study. Similar to the increase observed by [15] from 2.55 to 9.28 mg GAE/g following SSF, we also recorded a substantial rise in TPC: from 1.6 mg GAE/g in raw materials to 9.2 mg GAE/g in tempeh. However, it should be noted that our TPC values obtained using the Folin–Ciocalteu reagent did not correlate well with the more accurate LC-MS/MS measurements (Pearson’s correlation coefficient: r = 0.11, *p* = 0.604). This discrepancy is consistent with the observations in previous studies [2,6,8,53], where the Folin–Ciocalteu assay—widely used to quantify phenolic content in tempeh—has been shown to lack specificity. The reagent reacts not only with phenolic compounds but also with various non-phenolic substances, such as reducing sugars, ascorbic acid, and certain amino acids, leading to an overestimation of TPC values [54,55]. Therefore, implementing a sample clean-up step to remove these interfering compounds is recommended to obtain more representative TPC measurements. To our knowledge, no study has directly reported individual PAs in tempeh using LC-MS/MS; the existing data are limited to TPC values expressed as gallic acid equivalents (GAEs). When comparing our average TPC value in tempeh alternatives (9.7 mg GAE/g) with those reported for traditional soybean tempeh—14.6 mg GAE/g [56], 6.8 mg GAE/g [57], and 6.1 mg GAE/g [34]—it is evident that tempeh made from alternative raw materials falls within the typical TPC range observed in soybean-based tempeh.

As demonstrated by our findings and those of other researchers, the processes of soaking, dehulling (polishing), and cooking raw materials for tempeh production reduce the levels of PAs, although this loss can be partially offset by the release of bound PAs during SSF. Traditionally, removing the hulls is considered essential for optimal fermentation, texture, and digestibility. The hulls act as a physical barrier that impedes the growth of *Rhizopus* spp., while dehulled seeds allow for more uniform fermentation, a cohesive texture, improved digestibility, reduced bitterness (due to tannins), and faster cooking times. However, with growing emphasis on the health benefits of dietary fiber—particularly its positive impact on gut microbiota—future research should focus on developing processing technologies that retain seed hulls in tempeh made from alternative raw materials or soybeans. The goal should be to produce tempeh with comparable quality to that made from dehulled seeds while enhancing its nutritional profile by keeping the beneficial fiber and phenolic compounds in tempeh.

## 5. Conclusions

This study demonstrated the feasibility of producing tempeh from a variety of alternative plant sources, including pseudocereals (quinoa, buckwheat), legumes (yellow pea, white bean), and cereal grains (sorghum, proso millet). Using LC-MS/MS, we quantified seventeen phenolic acids and two flavan-3-ols (precursors of tannins) in both raw materials and tempeh alternatives in their free, conjugated, and insoluble forms. Solid-state fermentation increased the total phenolic acid content by an average of 11.3%, reaching 160.6 µg/g, predominantly in the conjugated and insoluble phenolic acid fractions. The highest total phenolic acid contents were found in tempeh samples containing sorghum, namely quinoa:sorghum (2:1) with 293 µg/g, sorghum:yellow pea (2:1) with 277.6 µg/g, and buckwheat:sorghum (1:1) with 271 µg/g. The most abundant phenolic acids were ferulic (18 µg/g), vanillic (14.6 µg/g), 3,4-dihydroxybenzoic (8 µg/g), and caffeic (6.7 µg/g). Our study has shown that tempeh made from alternative non-soy substrates offers a nutritious and allergen-friendly option to traditional soybean tempeh, particularly for consumers seeking plant-based, gluten-free, and GMO-free products. Future research should explore methods to retain seed hulls during processing to increase both fiber and phenolic content without compromising fermentation and sensory quality.

## Figures and Tables

**Table 1:** Phenolic acids in raw materials and tempeh.

	Raw Materials [µg/g]				Tempeh [µg/g]			
	Free	Conjugated	Insoluble	Total	Free	Conjugated	Insoluble	Total
Mean	28.02 ^a^	28.62 ^a^	87.65 ^b^	144.29 ^c^	21.22 ^f^	33.77 ^a^	105.62 ^d^	160.61 ^e^
STD	41.83	24.60	44.13	42.33	20.17	28.08	63.44	74.80
Min	0.76	3.36	22.71	50.88	3.52	4.47	17.08	52.35
Max	127.67	75.49	165.17	179.20	75.49	127.67	223.87	293.01

Superscript letters denote statistical differences.

**Table 2:** Phenolic acids in tempeh and raw material samples.

Tempeh or Raw Material	Phenolic Acids [µg/g]				TPC
	Free	Conjugated	Insoluble	Total	[mg GAE/g]
Beans (white, dehulled, cooked)	11.10	3.36	36.42	50.88	17.07
Buckwheat (dehulled, cooked)	23.47	21.07	88.11	132.64	0.73
Millet (dehulled, cooked)	0.76	13.27	165.17	179.20	13.45
Pea (yellow, split, dehulled, cooked)	127.67	14.11	22.71	164.48	2.20
Quinoa (white, cooked)	4.70	75.49	96.67	176.86	11.83
Sorghum (dehulled, cooked)	3.78	14.94	111.68	130.40	10.57
Sorghum (dehulled, uncooked)	34.41	55.12	82.88	172.42	1.44
Bean tempeh	7.53	21.10	23.72	52.35	1.83
Buckwheat tempeh	18.28	31.58	97.58	147.44	2.19
Buckwheat:Sorghum 1:1 (*w*/*w*) tempeh	30.93	38.64	201.46	271.03	16.46
Buckwheat:Sorghum 1:2 (*w*/*w*) tempeh	75.49	32.10	98.75	206.35	11.02
Millet tempeh	9.45	41.32	151.50	202.27	6.38
Pea (yellow) tempeh	59.46	11.24	17.08	87.78	15.89
Quinoa tempeh	10.14	127.67	87.20	225.02	8.44
Quinoa:Sorghum 1:1 (*w*/*w*) tempeh	22.32	24.84	75.18	122.34	2.05
Quinoa:Sorghum 1:2 (*w*/*w*) tempeh	7.81	30.65	84.33	122.78	4.68
Quinoa:Sorghum 2:1 (*w*/*w*) tempeh	9.68	59.46	223.87	293.01	2.46
Sorghum tempeh	3.78	4.47	87.79	96.03	8.81
Sorghum:Bean 1:1 (*w*/*w*) tempeh	4.84	25.53	155.78	186.15	5.57
Sorghum:Bean 3:1 (*w*/*w*) tempeh	3.52	23.35	65.74	92.60	4.43
Sorghum:Millet 3:1 (*w*/*w*) tempeh	11.41	15.01	57.10	83.52	5.17
Sorghum:Millet 96:120 (*w*/*w*) tempeh	16.20	52.90	45.00	114.10	10.00
Sorghum:Yellow pea 1:1 (*w*/*w*) tempeh	24.84	16.64	120.99	162.48	13.10
Sorghum:Yellow pea 1:2 (*w*/*w*) tempeh	30.65	18.28	86.02	134.95	10.14
Sorghum:Yellow pea 2:1 (*w*/*w*) tempeh	32.73	30.93	213.97	277.63	0.75

The values represent the averages of three replicate measurements. TPCs: total phenolic compounds measured by Folin and Ciocalteu’s reagent; GAEs: gallic acid equivalents.

## Data Availability

The original contributions presented in the study are included in the article/Supplementary Materials. Further inquiries can be directed to the corresponding author.

## Supplementary Materials

The following supporting information can be downloaded at: https://www.mdpi.com/article/10.3390/foods14132273/s1, Figure S1: Photographs of a selection of finished tempeh blends with sorghum; Figure S2: An example of an LC-MS/MS chromatogram in TIC of analytical standards; Figure S3: An example of an LC-MS/MS chromatogram in TIC of quinoa:sorghum tempeh (1:2, *w*/*w*); Table S1: Retention time of analytes including internal standard (IS) in alphabetical order; Text S1: LC-MS/MS method validation for phenolic acids in non-soy tempeh.

## Abbreviations

The following abbreviations are used in this manuscript:

ACS	American Chemical Society (grade of chemical purity)
BHA	Butylated Hydroxyanisole
DW	Dry Weight
EDTA-K_2_	Ethylenediaminetetraacetic Acid Disodium Salt Dihydrate
FW	Fresh Weight
GAEs	Gallic Acid Equivalents
GMO	Genetically-Modified Organism
HPLC	High-Performance Liquid Chromatography
IS	Internal Standard
LC-MS/MS	Liquid Chromatography Coupled with Tandem Mass Spectrometry
LOD	Limit of Detection
MRM	Multiple Reaction Monitoring
PA	Phenolic Acid
PP	Polypropylene
PTFE	Polytetrafluoroethylene
SD	Standard Deviation
SSF	Solid-State Fermentation
TIC	Total Ion Chromatogram
TPA	Total Phenolic Acid
TPC	Total Phenolic Content (by Folin–Ciocalteau reagent)
USD	United States Dollar
UV/VIS	Ultraviolet/Visible (spectrophotometry)
rpm	Revolutions per Minute
*v*/*v*	Volume per Volume
*w*/*v*	Weight per Volume

## Appendix A

**Table A1:** LC-MS/MS analysis of raw materials and tempeh alternatives. Four phenolic acids were not detected in any of the samples: shikimic acid, 3-hydroxycinnamic acid, 3,5-dihydroxybenzoic acid, and 2-hydroxycinnamic acid. Therefore, they are not included in the table. The values represent the averages of three replicate measurements. Empty cells indicate that the analyte was below the limit of detection (LOD). Abbreviated analytes: 3,4-B: 3,4-dihydroxybenzoic acid; 4-B: 4-hydroxybenzoic acid; 2,5-B: 2,5-dihydroxybenzoic acid; 3-B: 3-hydroxybenzoic acid; 4-C: 4-hydroxycinnamic acid.

Sample	Type	Gallic Acid	3,4-B Acid	Chlorogenic Acid	(+)-catechin	4-B Acid	2,5-B Acid	Caffeic Acid	Vanillic Acid	(−)-epicatechin	Syringic Acid	3-B Acid	4-C Acid	Sinapic Acid	Ferulic Acid	Salicylic Acid
Beans (white, dehulled, cooked)	free					4.32	0.05						2.19		3.23	1.30
	conjugated					0.17							0.81		2.39	
	insoluble	0.03	0.37			5.40	0.05	1.91					8.33	3.68	16.34	0.29
	total	0.03	0.37			9.89	0.10	1.91					11.33	3.68	21.97	1.59
Buckwheat (dehulled, cooked)	conjugated	2.66	2.43	0.16	7.50	1.89	0.09			4.59			0.25			3.89
	free	3.36	2.71			3.73	0.04	1.74					3.74	2.69	1.01	2.06
	insoluble	20.12	7.99		8.45	16.88	0.57	4.87	1.65	2.65	0.44	0.07	6.27	5.21	3.71	9.24
	total	26.14	13.13	0.16	15.95	22.50	0.70	6.61	1.65	7.24	0.44	0.07	10.26	7.90	4.72	15.19
Millet (dehulled, cooked)	free					0.34	0.01								0.41	
	conjugated		0.19			1.15	0.01	0.75					1.32		9.81	0.05
	insoluble	0.04	0.51			1.46	0.03	1.66	3.25		0.69		4.43	0.47	152.49	0.15
	total	0.04	0.70			2.94	0.05	2.41	3.25		0.69		5.74	0.47	162.71	0.20
Pea (yellow, split, dehulled, cooked)	free		4.42			9.85		0.85	73.54				6.20	1.03	31.78	
	conjugated		0.20			0.79	0.03	0.22					1.14	2.20	9.49	0.03
	insoluble	0.05	1.14			2.50	0.04	0.48					2.01	5.32	10.97	0.21
	total	0.05	5.77			13.14	0.07	1.55	73.54				9.34	8.55	52.24	0.24
Quinoa (white, cooked)	free					1.12	0.28		2.39				0.26		0.65	
	conjugated		19.25			9.99	0.35	7.90	18.68				3.53	0.65	14.92	0.22
	insoluble	0.02	0.49			13.21	1.98	2.95					11.73	0.78	65.24	0.26
	total	0.02	19.74			24.32	2.62	10.85	21.06				15.52	1.43	80.82	0.48
Sorghum (dehulled, cooked)	free		2.52		0.49	0.33	0.02	5.38					1.77		4.42	0.00
	conjugated		1.83	0.05	0.16	1.20	0.02	0.43								0.09
	insoluble	0.30	43.70		11.41	2.76	0.03	28.06	1.40	0.89	1.22		19.57	2.18		0.17
	total	0.30	48.05	0.05	12.06	4.29	0.07	33.86	1.40	0.89	1.22		21.34	2.18	4.42	0.26
Sorghum (dehulled, uncooked)	free		21.29	0.64	5.64	1.26	0.04	3.37					1.08		1.09	
	conjugated		6.38		1.36	1.22	0.02	26.56					5.96		13.52	0.10
	insoluble	0.17	35.30		5.97	1.95	0.02	17.69	1.23	0.46	0.51		18.31	1.12		0.16
	total	0.17	62.97	0.64	12.96	4.43	0.07	47.63	1.23	0.46	0.51		25.35	1.12	14.60	0.26
Bean tempeh	free					1.88	0.12						2.13		2.95	0.45
	conjugated		0.65			0.36	0.23	1.03					2.74	3.33	12.09	0.68
	insoluble	0.10	0.75			0.90	0.35	1.07				0.08	3.72	2.75	13.66	0.32
	total	0.10	1.41			3.14	0.70	2.10				0.08	8.59	6.08	28.71	1.45
Buckwheat tempeh	free		0.99		0.08	1.25	0.10	3.01					1.52	1.69	9.54	0.11
	conjugated	4.04	1.40		2.01	2.62	0.89	7.62		0.34			5.80	4.22	2.12	0.51
	insoluble	15.83	7.73		0.34	22.07	11.53	12.16		0.15			9.91	7.11	5.90	4.86
	total	19.87	10.11		2.44	25.93	12.52	22.78		0.50			17.23	13.02	17.56	5.48
Buckwheat:Sorghum 1:1 (*w*/*w*) tempeh	free		2.15		0.52	1.41	0.00	8.90					2.83	2.49	12.58	0.06
	conjugated	2.13	2.29		1.77	3.13	0.42	12.76		0.49			6.04	3.82	5.26	0.53
	insoluble	15.96	16.15		0.54	13.45	4.46	16.61		0.20			13.77	3.86	113.08	3.39
	total	18.09	20.58		2.83	17.99	4.88	38.27		0.69			22.63	10.17	130.91	3.98
Buckwheat:Sorghum 1:2 (*w*/*w*) tempeh	free		19.25			9.99	0.35	7.90	18.68				3.53	0.65	14.92	0.22
	conjugated	1.20	2.16		1.80	2.05		11.70		0.15			4.54	2.11	6.23	0.16
	insoluble	11.93	21.77		0.15	12.10	4.52	23.17			0.83		16.52	4.28		3.49
	total	13.13	43.18		1.96	24.14	4.87	42.77	18.68	0.15	0.83		24.58	7.04	21.16	3.87
Millet tempeh	free					1.05	0.05								8.27	0.08
	conjugated		1.45			0.77	0.18	8.40					2.62		27.83	0.08
	insoluble	0.08	0.45			2.34	0.09	1.38	3.94		0.36		4.44	0.66	137.61	0.15
	total	0.08	1.90			4.16	0.31	9.78	3.94		0.36		7.06	0.66	173.71	0.31
Pea (yellow) tempeh	free		4.20			4.33	0.21	5.22	23.93				3.14	0.82	17.52	0.08
	conjugated		0.41			1.03	0.09	0.19					0.80	1.84	6.75	0.13
	insoluble	0.05	1.05			4.33	0.59	0.32					0.91	3.22	6.23	0.37
	total	0.05	5.66			9.69	0.89	5.73	23.93				4.85	5.88	30.50	0.58
Quinoa tempeh	free		1.22			3.94	2.60						0.83		0.78	0.77
	conjugated		4.42			9.85		0.85	73.54				6.20	1.03	31.78	
	insoluble	0.05	0.64			6.89	0.97	1.40	14.59				3.05	0.38	59.04	0.18
	total	0.05	6.29			20.69	3.56	2.25	88.13				10.08	1.42	91.61	0.95
Quinoa:Sorghum 1:1 (*w*/*w*) tempeh	free		12.77	0.06		7.43	0.58	0.54					0.45		0.37	0.13
	conjugated								13.79				2.10	0.24	8.69	0.02
	insoluble	0.12	21.86			7.49	1.38	17.33	11.55				13.73	1.51		0.22
	total	0.12	34.63	0.06		14.92	1.95	17.87	25.34				16.29	1.75	9.05	0.36
Quinoa:Sorghum 1:2 (*w*/*w*) tempeh	free		5.24	0.05		1.62	0.35						0.21		0.20	0.14
	conjugated		4.89			1.91		5.60	7.95				2.63	0.53	7.12	0.02
	insoluble	0.12	25.88	0.00	0.10	5.74	1.07	20.57	10.61		0.73		17.66	1.62	0.00	0.22
	total	0.12	36.01	0.05	0.10	9.27	1.43	26.17	18.56		0.73		20.49	2.15	7.32	0.38
Quinoa:Sorghum 2:1 (*w*/*w*) tempeh	free		5.24			2.75	0.79						0.35		0.38	0.18
	conjugated		4.20			4.33	0.21	5.22	23.93				3.14	0.82	17.52	0.08
	insoluble	0.05	14.21			5.87	1.13	11.42	12.09				10.27	1.15	167.47	0.20
	total	0.05	23.65			12.95	2.13	16.64	36.02				13.76	1.98	185.36	0.46
Sorghum tempeh	free		1.83	0.05	0.16	1.20	0.02	0.43								0.09
	conjugated					0.26	0.02	0.44					0.91		2.73	0.12
	insoluble	0.37	29.62		5.72	3.56	0.08	23.93	1.46	0.48	0.83		19.49	2.05		0.20
	total	0.37	31.44	0.05	5.88	5.02	0.12	24.80	1.46	0.48	0.83		20.39	2.05	2.73	0.41
Sorghum:Bean 1:1 (*w*/*w*) tempeh	free		0.68			0.65	0.06	0.63					0.68		1.82	0.34
	conjugated		1.40			0.67	0.12	6.29					3.11	1.70	11.73	0.50
	insoluble	0.14	15.49			1.44	0.28	12.70	0.92		0.49	0.03	11.73	3.17	109.01	0.31
	total	0.14	17.57			2.76	0.46	19.62	0.92		0.49	0.03	15.52	4.87	122.56	1.14
Sorghum:Bean 3:1 (*w*/*w*) tempeh	free		0.88	0.09	0.22	0.58	0.03	0.52					0.24		0.62	0.33
	conjugated		1.89			0.58	0.04	8.36					2.74	1.37	7.81	0.55
	insoluble	0.17	23.62		0.17	1.88	0.15	18.34	1.27		0.65	0.08	16.48	2.70		0.19
	total	0.17	26.39	0.09	0.40	3.04	0.21	27.22	1.27		0.65	0.08	19.47	4.06	8.43	1.07
Sorghum:Millet 3:1 (*w*/*w*) tempeh	free		3.84	0.33		4.81	0.09	1.75							0.49	0.10
	conjugated		2.31			0.21		6.84					1.97		3.45	0.22
	insoluble	0.16	21.30		0.07	1.59	0.16	14.40	0.93		0.73		16.46	1.15		0.15
	total	0.16	27.46	0.33	0.07	6.62	0.25	22.99	0.93		0.73		18.43	1.15	3.94	0.47
Sorghum:Millet 96:120 (*w*/*w*) tempeh	free	0.09	1.80	0.11		3.65	0.06	1.01					0.21		9.21	0.06
	conjugated		2.58			0.80	0.20	17.09					4.23		27.95	0.05
	insoluble	0.14	15.61			1.93	0.23	10.89	2.30		0.61		12.03	1.10		0.15
	total	0.23	19.99	0.11		6.39	0.49	29.00	2.30		0.61		16.47	1.10	37.15	0.26
Sorghum:Yellow pea 1:1 (*w*/*w*) tempeh	free								13.79				2.10	0.24	8.69	0.02
	conjugated		1.40		0.23	1.19	0.07	3.27					1.30	1.47	7.63	0.08
	insoluble	0.07	13.71			3.89	0.38	8.97					13.38	2.95	77.31	0.34
	total	0.07	15.11		0.23	5.07	0.46	12.23	13.79				16.79	4.66	93.63	0.44
Sorghum:Yellow pea 1:2 (*w*/*w*) tempeh	free		4.89			1.91		5.60	7.95				2.63	0.53	7.12	0.02
	conjugated		0.99		0.08	1.25	0.10	3.01					1.52	1.69	9.54	0.11
	insoluble	0.06	8.59			3.35	0.60	6.48					5.92	3.44	57.23	0.34
	total	0.06	14.47		0.08	6.51	0.70	15.09	7.95				10.07	5.66	73.89	0.48
Sorghum:Yellow pea 2:1 (*w*/*w*) tempeh	free		5.31			2.04	0.00	6.45	7.59				2.89	0.44	7.95	0.05
	conjugated		2.15		0.52	1.41	0.00	8.90					2.83	2.49	12.58	0.06
	insoluble	0.10	18.27			3.59	0.45	14.83					14.03	3.26	159.19	0.25
	total	0.10	25.73		0.52	7.04	0.45	30.18	7.59				19.75	6.19	179.71	0.36

## Appendix B

**Table A2:** Mass spectrometric parameters for analytes. Q—quantitation ion; C—confirmation ion. For retention times, see Supplementary Materials.

Compound Name	Precursor Ion *m*/*z*	Product Ion *m*/*z*	Fragmentor Voltage	Collision Energy V
(−)-epicatechin C	289.1	109	111	28
(−)-epicatechin Q	289.1	245.1	111	12
(+)-catechin C	289.1	123	107	32
(+)-catechin Q	289.1	109	107	24
2,5-dihydroxybenzoic acid C	153	109	73	12
2,5-dihydroxybenzoic acid Q	153	108	73	20
2-hydroxycinnamic acid C	163	117	76	24
2-hydroxycinnamic acid Q	163	119	76	8
3,4-dihydroxybenzoic acid C	153	108	73	24
3,4-dihydroxybenzoic acid Q	153	109	73	12
3,5-dihydroxybenzoic acid C	153	67	70	16
3,5-dihydroxybenzoic acid Q	153	109	70	8
3-hydroxybenzoic acid Q	137	93	64	8
3-hydroxycinnamic acid C	163	91	67	24
3-hydroxycinnamic acid Q	163	119	67	12
4-hydroxybenzoic acid C	137	65.1	73	36
4-hydroxybenzoic acid Q	137	93	73	12
4-hydroxycinnamic acid C	163	119	76	8
4-hydroxycinnamic acid Q	163	119	67	12
Caffeic acid C	179	134	86	28
Caffeic acid Q	179	135	86	12
Chlorogenic acid C	353.1	85	89	48
Chlorogenic acid Q	353.1	191.1	89	12
Ferulic acid Q	193.1	134	73	16
Gallic acid C	169	79	86	24
Gallic acid Q	169	125	86	12
IS (3,5-dichloro-4-hydroxybenzoic acid) C	204.9	35	141	24
IS (3,5-dichloro-4-hydroxybenzoic acid) Q	204.9	161	141	12
Salicylic acid C	137	65.1	86	32
Salicylic acid Q	137	93	86	16
Shikimic acid C	173	111	113	4
Shikimic acid Q	173	93	113	12
Sinapic acid C	223.1	193	89	20
Sinapic acid Q	223.1	149	89	20
Syringic acid C	197	167	70	16
Syringic acid Q	197	123	70	24
Vanillic acid C	167	108	70	16
Vanillic acid Q	167	152	70	12
