# Supplementary material for: Quantification of Seventeen Phenolic Acids in Non-Soy Tempeh Alternatives Based on Legumes, Pseudocereals, and Cereals
Source: Foods. 2025 Jun 26;14(13):2273. doi: 10.3390/foods14132273 (PMC12248807; doi:10.3390/foods14132273)
Supplement: Supplementary file 1 [file foods-14-02273-s001.zip › foods-3725758-supplementary.pdf]

Supplementary material

MDPI-Foods

Article

Quantification of Seventeen Phenolic Acids in Non-Soy Tempeh Alternatives Based on Legumes, Pseudocereals, and Cereals

By Miloslav Šulc and Jana Rysová

Contents:

Figure S1: Photographs of selected tempeh blends with sorghum 2
Figure S2: An example of an LC-MS/MS chromatogram of analytical standards 2
Figure S3: An example of an LC-MS/MS chromatogram of non-soy tempeh 3
Table S1: Retention time of analytes 3
Text S1: LC-MS/MS validation data for sorghum tempeh 4

Figure S1. Photographs of a selection of finished tempeh blends with sorghum

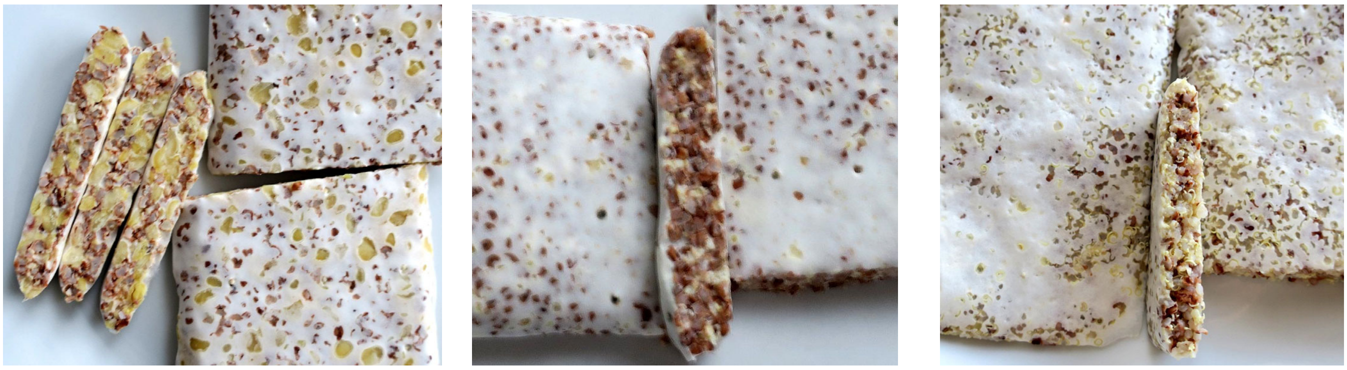

A B C

A – sorghum:yellow pea (1:1, w/w); B – sorghum:proso millet (3:1, w/w); C – quinoa:sorghum (2:1, w/w).

Figure S2: An example of an LC-MS/MS chromatogram in TIC of analytical standards

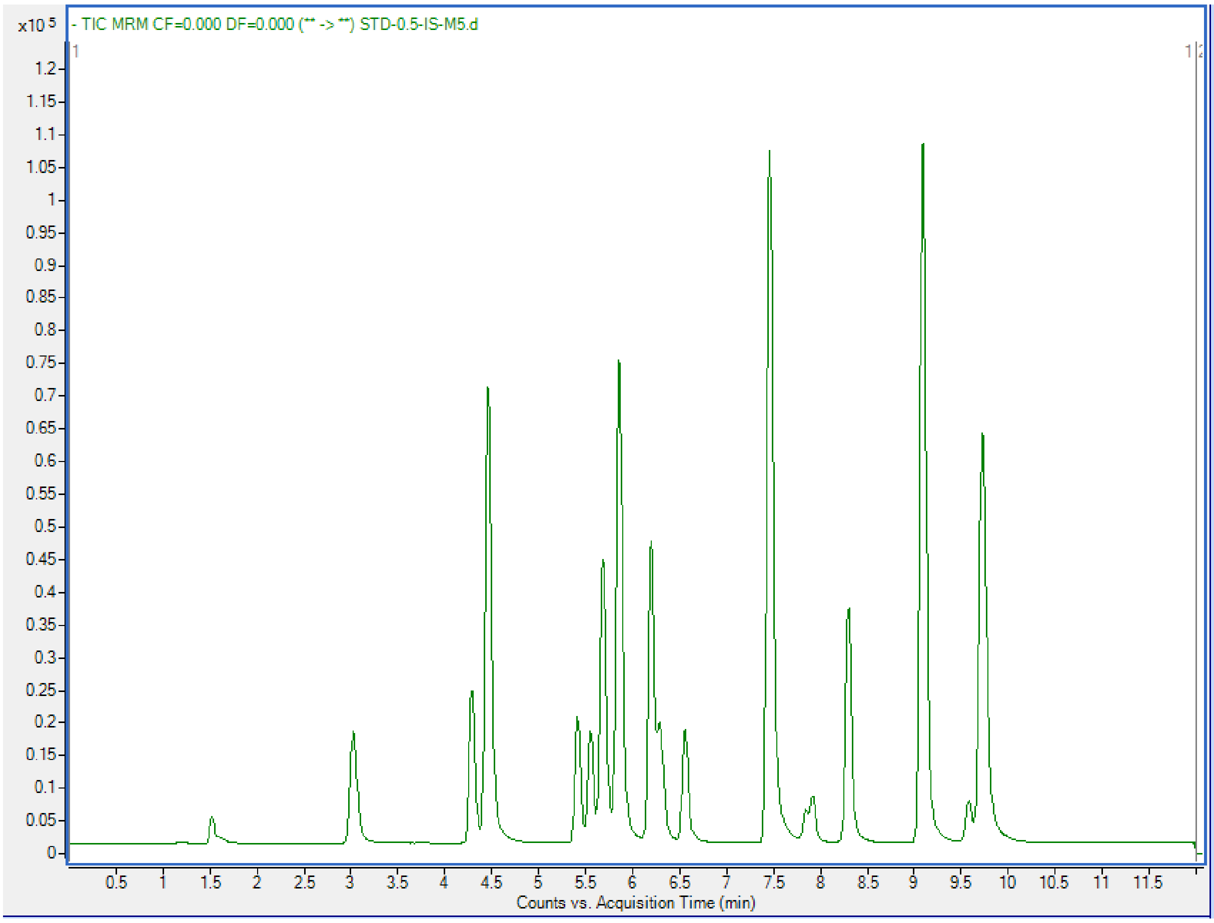

For compounds and their retention time see Table A (page 3)

Figure S3: An example of an LC-MS/MS chromatogram in TIC of quinoa:sorghum tempeh (1:2, w/w)

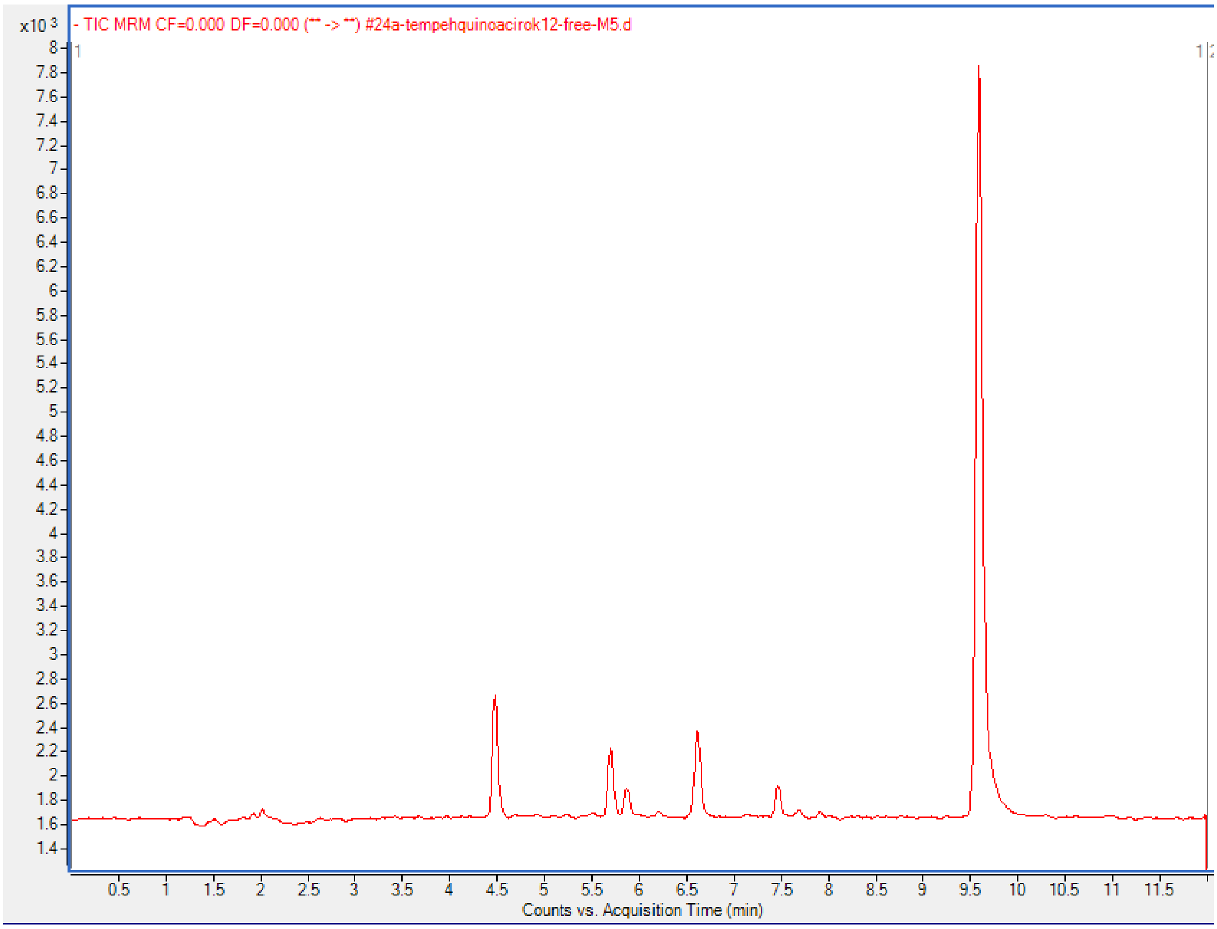

For compounds and their retention time see Table A (page 3)

Table S1: Retention time of analytes including internal standard (IS) in alphabetical order

Analyte	Retention time [min]	Analyte	Retention time [min]
(+)-catechin	5.54	Caffeic acid	6.19
(-)-epicatechin	6.28	Chlorogenic acid	5.40
2,5-dihydroxybenzoic acid	5.85	Ferulic acid	7.90
2-hydroxycinnamic acid	9.08	Gallic acid	3.00
3,4-dihydroxybenzoic acid	4.45	IS (3,5-dichloro-4-hydroxybenzoic acid)	9.55
3,5-dihydroxybenzoic acid	4.27	Salicylic acid	9.72
3-hydroxybenzoic acid	6.55	Shikimic acid	1.52
3-hydroxycinnamic acid	8.28	Sinapic acid	7.83
4-hydroxybenzoic acid	5.68	Syringic acid	6.32
4-hydroxycinnamic acid	7.44	Vanillic acid	6.19

Text S1: LC-MS/MS method validation for phenolic acids in non-soy tempeh

Validation data for phenolic acids in a non-soy tempeh matrix were generated through a single-laboratory validation study, involving two analysts working independently on six separate occasions (2 analysts × 3 days) over a six-month period in the latter half of 2023. The study was conducted using a single 500 g batch of in-house prepared sorghum tempeh, with validation performed separately for the free, conjugated, and insoluble phenolic acid fractions.

Sample preparation, including defatting, extraction steps, and the use of an internal standard, is detailed in Section 2 (Materials and Methods) of the main manuscript. Analytical measurements were performed in duplicate for each matrix type. Due to the absence of a suitable blank matrix, method validation was carried out on samples spiked with 50% of the intrinsic concentration of each phenolic acid.

Injection repeatability of the LC-MS/MS system was consistently below 1.2% relative standard deviation (RSD) for peak areas across all analytes, based on six consecutive injections during the entire validation period. As blank matrices were not available, limits of detection (LOD) could not be established via spiking experiments. Instead, LOD and limit of quantification (LOQ) were estimated based on signal-to-noise (S/N) ratios of 3:1 and 10:1, respectively, using serial dilutions of standard solutions until target S/N thresholds were achieved. The LOQ was defined as the lowest concentration within the established linear working range.

Owing to the lack of certified reference materials, method trueness and recovery could not be rigorously assessed. Calibration curves for all analytes exhibited the best fit with a quadratic regression model, with the lowest coefficient of determination (R^2) observed during the validation being 0.9375. Representative validation data are presented for four phenolic acids, which were consistently detected in sorghum tempeh in all three forms: free, conjugated, and insoluble.

Free phenolic acids

	PA content [µg/g]	Repeatability CV(r) [%]	Intermediate reproducibility CV(iR) [%]	Intermediate reproducibility iR [%]	Recovery [%], estimated
4-hydroxybenzoic ac.	2.01	3.7	5.2	8.6	94.5
2,5-hydroxybenzoic ac.	0.032	7.8	9.2	17.8	82.3
Caffeic ac.	0.69	4.7	5.1	8.9	93.9
Salicylic acid	0.152	4.4	5.7	10.2	90.1

Conjugated phenolic acids

	PA content [µg/g]	Repeatability CV(r) [%]	intermediate reproducibility CV(iR) [%]	Intermediate reproducibility iR [%]	Recovery [%], estimated
4-hydroxybenzoic ac.	0.32	5.1	7.2	11.9	94.8
2,5-hydroxybenzoic ac.	0.028	8.6	9.9	20.4	84.7
Caffeic ac.	0.47	5.6	7.1	12.3	96.3
Salicylic acid	0.19	6.3	7.8	11.5	89.5

Bound phenolic acids

	PA content [μg/g]	Repeatability CV(r) [%]	intermediate reproducibility CV(iR) [%]	Intermediate reproducibility iR [%]	Recovery [%], estimated
4-hydroxybenzoic ac.	5.43	4.9	5.9	9.6	93.2
2,5-hydroxybenzoic ac.	0.106	7.5	8.7	14.7	89.9
Caffeic ac.	29.14	4.1	6.3	9.9	97.8
Salicylic acid	0.31	6.2	7.8	13.7	92.1

Estimation of LOD and LOQ by dilution of sample matrix

	LOD [μg/g]	LOQ [μg/g]
4-hydroxybenzoic ac.	0.0018	0.006
2,5-hydroxybenzoic ac.	0.0071	0.025
Caffeic ac.	0.0043	0.014
Salicylic acid	0.0135	0.041
